# Efficacy of Biodegradable Curcumin Nanoparticles in Delaying Cataract in Diabetic Rat Model

**DOI:** 10.1371/journal.pone.0078217

**Published:** 2013-10-14

**Authors:** Charitra N. Grama, Palla Suryanarayana, Madhoosudan A. Patil, Ganugula Raghu, Nagalla Balakrishna, M. N. V. Ravi Kumar, Geereddy Bhanuprakash Reddy

**Affiliations:** 1 Strathclyde Institute of Pharmacy and Biomedical Sciences, University of Strathclyde, Glasgow, United Kingdom; 2 National Institute of Nutrition, Hyderabad, India; Duke University, United States of America

## Abstract

Curcumin, the active principle present in the yellow spice turmeric, has been shown to exhibit various pharmacological actions such as antioxidant, anti-inflammatory, antimicrobial, and anti-carcinogenic activities. Previously we have reported that dietary curcumin delays diabetes-induced cataract in rats. However, low peroral bioavailability is a major limiting factor for the success of clinical utilization of curcumin. In this study, we have administered curcumin encapsulated nanoparticles in streptozotocin (STZ) induced diabetic cataract model. Oral administration of 2 mg/day nanocurcumin was significantly more effective than curcumin in delaying diabetic cataracts in rats. The significant delay in progression of diabetic cataract by nanocurcumin is attributed to its ability to intervene the biochemical pathways of disease progression such as protein insolubilization, polyol pathway, protein glycation, crystallin distribution and oxidative stress. The enhanced performance of nanocurcumin can be attributed probably to its improved oral bioavailability. Together, the results of the present study demonstrate the potential of nanocurcumin in managing diabetic cataract.

## Introduction

It is estimated that currently around 366 million people worldwide have diabetes and projected to rise to 552 million in 2030 [1]. Uncontrolled or poorly controlled diabetes can lead to micro- (eg. blindness, nephropathy and neuropathy) and macrovascular (cardiovascular and stroke) complications [2]. Blindness due to cataract is an important long-term complication of diabetes. Cataract, characterized by cloudiness or opacification of the crystalline eye lens, is the leading cause of blindness all over the world and more so in developing countries [3]. Many studies indicate the role of oxidative stress in mediating micro- and macrovascular complications due to glucose autoxidation, non-enzymatic glycation of proteins leading to advanced glycation end products (AGEs), and enhanced glucose flux through polyol pathway [4,5]. Though the etiology of cataract is not fully understood, oxidative damage to the constituents of the eye lens is considered to be a major mechanism in the initiation and progression of various types of cataracts, including diabetic cataract [6]. This research has prompted the use of dietary antioxidant in an attempt to slow the cataract progression [7–9]. 

In this context, our group has evaluated a number of traditional medicines and common dietary agents for their potential to delay diabetic cataract [10–13]. Based on these studies, we found that curcumin, active principle found in yellow spice turmeric, delays diabetes-induced cataract in rats. We have demonstrated that feeding of dietary antioxidant curcumin was effective in delaying streptozotocin (STZ)-induced diabetic cataract in rats mainly through its antioxidant property [12]. In addition, we also found that curcumin inhibited diabetes-induced expression of vascular endothelial growth factor (VEGF) in rat retina [14] and lens aldose reductase (AR) [15]. The dose used (0.01%) in the study [12] is equivalent or slightly higher than the amount of average daily intake (ADI) of turmeric [16].

Though dietary supplements such as curcumin offered some benefits in preclinical studies, the translation has been very poor and the doses used in clinical trials are unfeasible to practice in reality. One of the major reasons for the lack of clinical success with curcumin is linked to its extensive intestinal and hepatic metabolic biotransformation resulting in poor oral bioavailability [17–20]. Recently, the focus is to address bioavailability concerns of the supplements with a view to improve the therapeutic efficacy [21]. Use of polymeric nanoparticles has been actively explored as oral delivery vehicles for pharmaceutically challenging compounds [22–24]. We have shown that encapsulating curcumin in polymer nanoparticles improved peroral bioavailability of curcumin at least 9 times compared to that of plain curcumin in rodents [25]. The present study reports the efficacy of curcumin encapsulated nanoparticles in delaying cataract in diabetic rat model. 

## Materials and Methods

### Materials

Streptozotocin (STZ), curcumin, NADPH, NAD, 2-thiobarbituric acid, 1,1,3,3-tetraethoxy propane (TEP), DL-glyceraldehyde, lithium sulfate, β-mercaptoethanol, sorbitol, sorbitol dehydrogenase, BSA, 2,4-dinitrophenylhydrazine, pyrogallol, diethylenetriaminepentaacetic acid and EDTA were obtained from Sigma-Aldrich (St. Louis, MO). Polyvinyl alcohol (PVA) (MW 30–70 kDa) and ethyl acetate were purchased from Sigma-Aldrich (Poole, UK). Poly(lactide-co-glycolic) acid (PLGA) (Resomer R503H; MW 35–40 kDa) was purchased from Boehringer Ingelheim, (Ingelheim, Germany). Curcumin was purchased from Indsaff, Punjab, India.

### Preparation and characterization of curcumin encapsulated PLGA nanoparticles

The particles were prepared by a modified emulsion-diffusion-evaporation method previously developed in our laboratory [25]. Curcumin (7.5 mg) and polymer, PLGA (50 mg), were dissolved in 2.5 ml of ethyl acetate and allowed to stir at 1000 rpm for 1 h to obtain homogeneous solution and so was 50 mg of PVA in 5 ml of distilled water. The organic phase was then added in a drop wise manner under stirring to the PVA solution and stirring continued for 5 min. The emulsion was homogenized for 5 min at 15,000 rpm and transferred to 20 ml water to facilitate diffusion of ethyl acetate and was stirred at 1000 rpm overnight to ensure the complete evaporation of ethyl acetate and to get the nanoparticles. The nanoparticles suspension was then centrifuged at 14,000g for 30 min to separate unbound PVA and curcumin from the preparation. The pellet obtained was then re-suspended in required amount of distilled water using mild sonication for 10 sec. The particle size (fresh preparation) at this stage was measured using zeta sizer. The re-suspended pellet was freeze dried (2 ml suspension in a 5 ml vial) using 5% sucrose (w/v) and stored for later studies. The freeze drying was performed using the conditions: freezing (8 h) at -50 °C; main drying (54 h) -50 °C, vacuum 0.0035 mBar, safety pressure 1.650 mBar and final drying main drying (9 h) +20°C, vacuum 0.0035 mBar, safety pressure 1.650 mBar. The freeze dried particles were re-suspended in 1 ml of water and characterized for size and entrapment efficiency. Blank particles were prepared and processed similarly without curcumin.

### Experimental design and dietary regimen

Three months old male WNIN rats with average body weight of 235 ± 15 g (obtained from the National Center for Laboratory Animal Sciences, National Institute of Nutrition, Hyderabad) were used for this study. All the animals were fed AIN-93 diet ad libitum throughout the study. The control (Control; n=8) rats received 0.1 M citrate buffer, pH 4.5 as vehicle and the experimental rats received a single intraperitoneal injection of STZ (32 mg/kg) in citrate buffer. After 72 h, blood glucose levels were monitored in rats fasted for 16 h. Animals with blood glucose levels <150 mg/dl were excluded from the experiment and rest were distributed into four groups. Animals in these groups received either only DMSO as vehicle, (Diabetic, D; n=9) or received 2 mg /day curcumin in DMSO, (D+curcumin; n=8), 2 mg/ day nanocurcumin in water (D+nanocurcumin; n=8) and only blank nanoparticles in water (D+blank particles; n=6) for a period of 10 weeks. Curcumin oral dose (2 mg/day) corresponds to approximately 0.01% curcumin in the diet as used in our previous study [12]. 

### Animal Care

Institutional and national guidelines for the care and use of animals were followed and all experimental procedures involving animals were approved by the IAEC (institutional animal ethical committee) of National Institute of Nutrition. Animals were housed in individual cages in a temperature (22°C) and humidity (50%) controlled room with a 12-h light/dark cycle. All the animals had free access to water. Food intake (daily) and body weights (weekly) were monitored. We adhered to the ARVO Statement for the Use of Animals in Ophthalmic and Vision Research.

### Slit lamp examination and Cataract grading

Eyes were examined every week using a slit lamp biomicroscope (Kowa SL15, Portable slit lamp, Tokyo, Japan) on dilated pupils. Initiation and progression of lens opacity was graded into five categories as described previously [10–13].

### Blood/ Lens collection and processing

Blood was drawn once in a week from retro orbital plexus for glucose and insulin estimation. At the end of 10 weeks, animals were sacrificed by CO_2_ asphyxiation and lenses were dissected by posterior approach and stored at -70°C until further analysis. Some of the biochemical estimations such as malondialdehyde (MDA), sorbitol cannot be done with two lenses of each rat as the rat lens weigh about 40 mg. Hence we pooled 3-5 lenses for making homogenate not only to estimate MDA and sorbitol but also for other biochemical estimations. A 10% homogenate was prepared from 3-5 pooled lenses in 50 mM phosphate buffer, pH 7.4. All the biochemical parameters were analyzed in the soluble fraction of the lens homogenate (15,000x g at 4°C) except for lens malondialdehyde (MDA) and sorbitol, which was determined in the total homogenate.

### Biochemical estimations

Serum glucose was measured by GOD-POD method using a kit (BioSystems S.A.Costa Brava 30, Barcelona, Spain) and serum insulin by a radioimmuno assay (RIA) kit (BRITE-DAE, Mumbai, India) according to the manufacturer’s instructions. Lens MDA, as thiobarbituric acid reacting substances (TBARS), protein carbonyl content, and activities of aldose reductase (AR) and sorbitol levels were determined according to the methods described previously[26]. The specific activity of superoxide dismutase (SOD) was assayed according to the reported methods [26]. Total, soluble and insoluble protein was assayed by Lowry method using BSA as standard. 

### SDS-PAGE and size exclusion chromatography of lens proteins

Subunit profile and cross-linking of soluble proteins were analyzed on 12% polyacrylamide in the presence of SDS under reducing conditions[26]. In brief, protein samples (20µg) and molecular mass markers were resolved on a 12% polyacrylamide gel. The gel was stained with staining solution (methanol, glacial acetic acid and water in 1:6:6) containing 0.25% coomassie-R250 and the protein bands were visualized after distaining with methanol, glacial acetic acid and water in (1:6:6). Crystallin distribution in the soluble protein fraction was assessed by size exclusion chromatography on a 600x7.5 mm TSK-G3000 SW column (TOSOH Co., Japan) using a Shimadzu Class-VP HPLC system [12,13]. The column was equilibrated with 0.1 M sodium phosphate buffer pH 6.7 containing 0.1 M sodium chloride at a flow rate of 1 ml/min. Soluble protein samples (20μl of 1 mg/ml solution) were loaded onto the column and protein peaks were detected at 280 nm.

### Statistical analysis

SPSS version 19.0 software was used for statistical analysis. Descriptive statistics was calculated for all variables. One-way ANOVA was used for comparison of mean values across groups and multiple comparisons were made by Duncan’s multiple range test. Whenever heterogeneity of variance was found, non-parametric Mannwhitney “U” test was carried out. Linear regression analysis was carried out to study the relationship for cataract progression with the duration (weeks) and groups (dummy variables). A *p*-value <0.05 was considered significant.

## Results

### Preparation of and characterization of curcumin encapsulated PLGA nanoparticles

**Figure 1:**
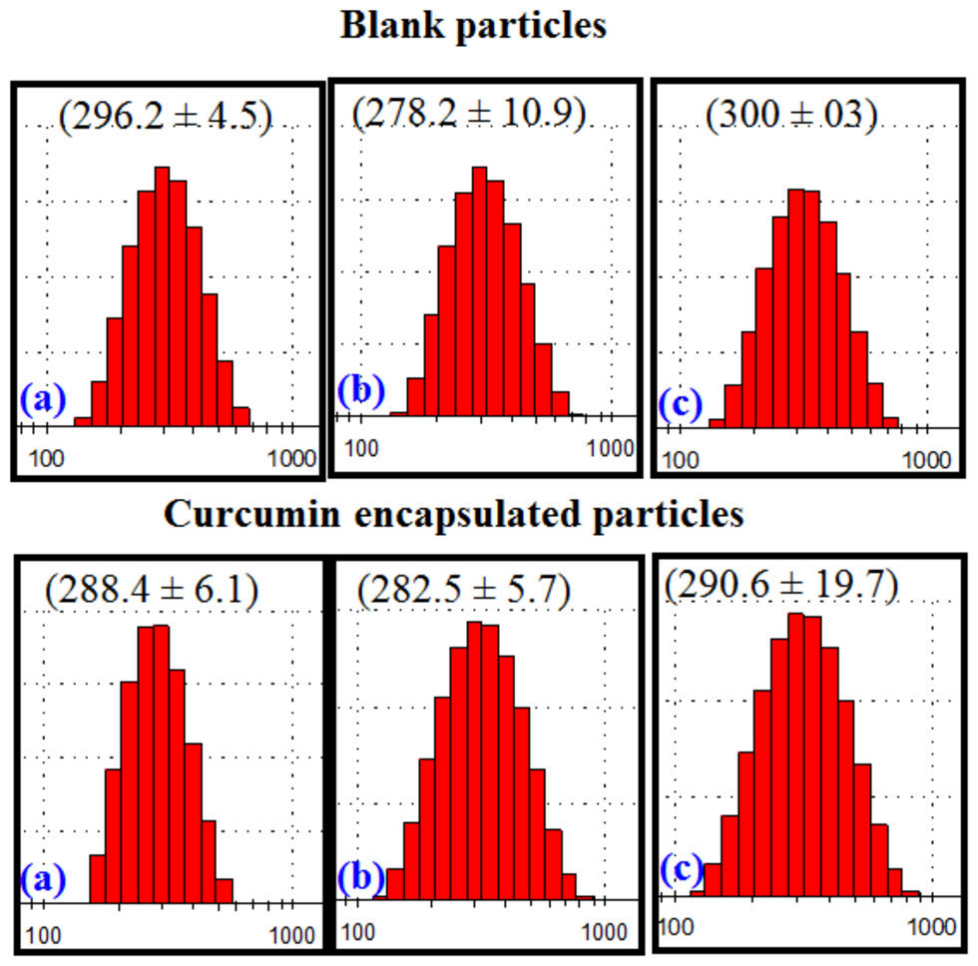
Particle characteristics measured by Malvern zeta sizer fresh (A) after centrifugation (B) and after freeze drying (C). The numbers in parenthesis are average particle size in nm.

Curcumin nanoparticles were prepared successfully by previously developed emulsion-diffusion-evaporation technique with slight modification [25]. The Z-average particle size of nanoparticles at 7.5 mg curcumin loading was 282.50±5.72 nm with PDI of 0.14±0.06 resulting in approximately 4.2 mg of curcumin encapsulated (56%) in the particles (Figure 1). The size of blank particles without curcumin was 281.3 ± 8.8 nm with 0.07 ± 0.01 PDI. The freeze dried curcumin loaded and blank particles were comparable to the respective fresh preparations.

### Food intake and body weights

As observed in previous studies [10–13], the food intake of diabetic animals was more than the respective controls. Despite the increased food intake, the body weight of diabetic animals decreased significantly (mean ± S.E body weight at the end of experiment, 205 ± 4.35 g; p<0.001) when compared to control animals (320 ± 3.65 g) and so was the case in the treated groups, curcumin, nanocurcumin and blank particle groups [202 ± 3.15 g, p<0.001; 198±5.22 g, p<0.001 and 201± 2.34 g, p<0.001, respectively].

### Cataract progression

**Figure 2:**
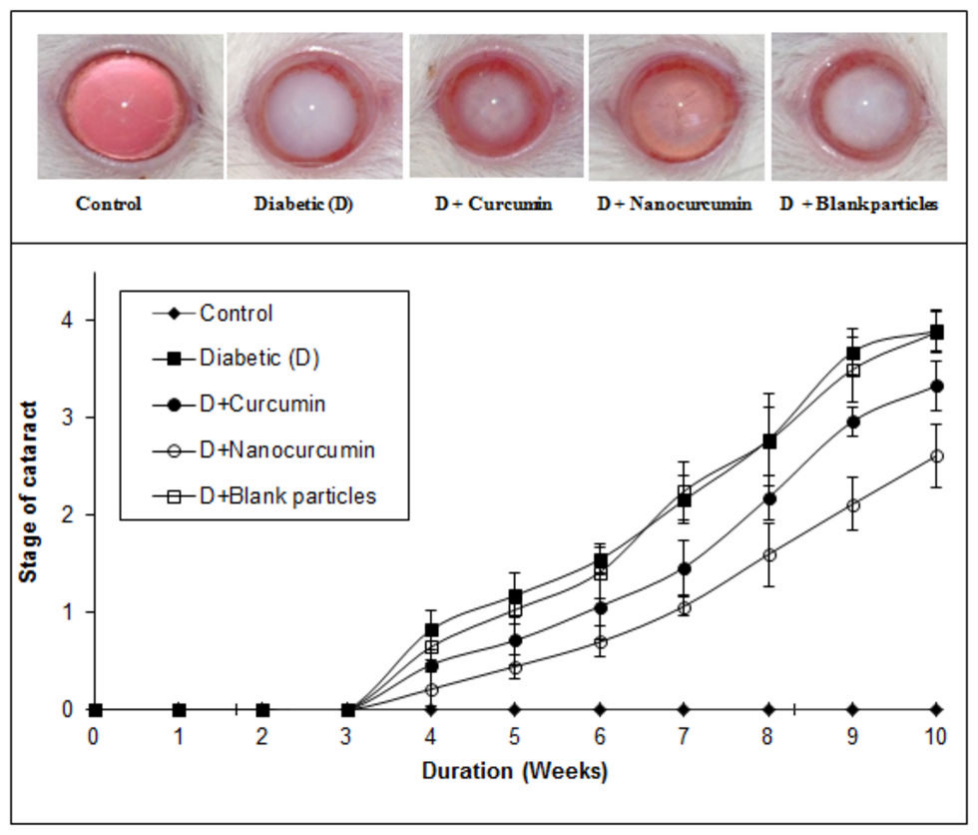
The effect of curcumin and nanocurcumin on STZ-induced cataract in rats. *Top* *panel*: Representative photographs of lens from each group at the end of 10 weeks. *Bottom* *panel*: Quantitative representation of cataract progression in each group with time. Stage of cataract in each group was averaged at a given time and the average stage of cataract along with standard error was plotted as a function of time.

While the onset of cataract due to hyperglycemia was observed in diabetic animals after four weeks of STZ injection and progressed to mature cataract by eight weeks in untreated diabetic group, feeding of dietary curcumin delayed both onset and progression of cataract (β= -0.379; SE=0.080) as compared to untreated group (Figure 2) and this is consistent with our previous observations [12]. Since lenses were in different stages of cataract in a given group at a given time, we have averaged the stages at the given time for a given group, to see the onset and progression of cataract in an empirical manner in all the groups and presented with duration of diabetes (Figure 2). Interestingly, there was a further and significant (p<0.001) delay in the onset and progression of cataract in nanocurcumin group (β= -0.298; SE=0.091) as compared to dietary curcumin diabetic group. At the end of ten weeks, the severity of cataract was significantly (p<0.001) lower in nanocurcumin group compared to untreated as well as dietary curcumin fed diabetic rats. All the lenses in control group appeared to be normal and free of opacities during the experimental period, on the other hand, blank nanoparticles did not affect the onset and progression of cataract(β= -0.570; SE=0.080). 

### Blood glucose and insulin levels

**Figure 3:**
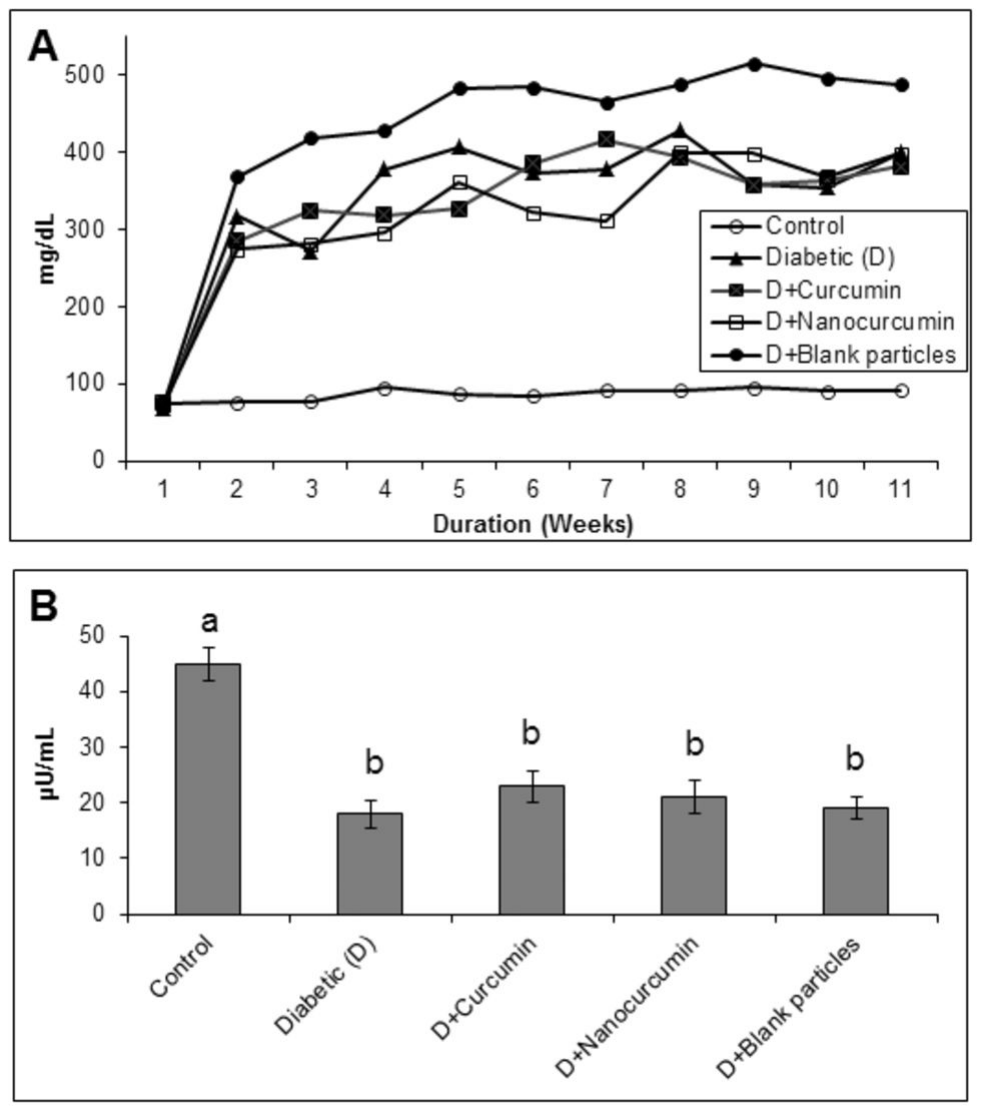
The effect of curcumin and nanocurcumin on blood glucose (Panel A) and insulin levels (Panel B) in STZ-treated rats. Data are average of all the animals in a given group. Different superscripts in Panel B indicate data are significantly different from other groups.

The blood glucose and insulin levels were estimated to understand if the role of curcumin in cataract onset and progression is due to reduction of STZ-induced hyperglycemia. As expected blood glucose levels were elevated and insulin levels were lowered significantly in untreated group compared to the controls (Figure 3). However, both forms of curcumin failed to reduce glucose concentrations and increase insulin levels (Figure 3) indicating that curcumin’s role in delaying cataract progression is independent of its glucose lowering ability. 

### Molecular basis for the delay of cataract

To investigate the effect of curcumin on possible mechanisms related to delay of diabetic cataract, the major biochemical parameters related to cataractogenesis such as oxidative stress/antioxidant system, polyol pathway, protein glycation, protein oxidation, protein content, and crystallin distribution were studied. 

### Oxidative stress & antioxidant system

Though statistically not significant, the rise in MDA levels and protein carbonyl content in diabetic untreated group compared to control group indicates increased lipid peroxidation and enhanced protein oxidation in the lens (Table 1). Further, altered activity of antioxidant enzyme SOD (Table 1) suggests an increased oxidative stress in diabetic cataract lens. Both the forms of curcumin prevented the alterations in MDA and protein carbonyls despite elevated glucose levels, but not in SOD levels (Table 1). Again the nanocurcumin was more pronounced with respect to MDA levels and protein carbonyl content, though the difference is statistically insignificant.

**Table 1:** The effect of curcumin and nanocurcumin on malondialdehyde (MDA), protein carbonyls and superoxide dismutase (SOD) parameters in rat lens.

**Groups**	**MDA**	**Protein carbonyls**	**SOD**
Control	6.53 ± 2.139	5.87 ± 0.617	53.03 ± 0.54
Diabetic (D)	9.88 ± 1.371	7.04 ± 1.764	62.18 ± 4.39
D + Curcumin	8.19 ± 0.661	6.49 ± 1.462	68.16 ± 9.54
D + Nanocurcumin	7.40 ± 1.577	6.15 ± 1.549	66.42 ± 15.51
D + Blank particles	8.44 ± 1.235	7.05 ± 0.308	62.57 ± 2.46
	p=0.121	p=0.680	p=0.291

The data are mean ± SD (n=4). MDA levels are expressed as n moles/g lens, protein carbonyls as n moles/mg protein and SOD activity as units/min/100 mg protein.

### Polyol pathway

While the specific activity of AR, a key enzyme of the polyol pathway was higher, but statistically insignifican , in diabetic animals than in control, the increase in sorbitol, product of AR catalyzed reaction, was remarkably high (Table 2). Treatment with curcumin resulted in normalization of AR activity and the effect was prominent with nanocurcumin (Table 2). While, the levels of sorbitol were decreased with curcumin, the reduction was highly significant (p<0.001) with nanocurcumin (Table 2). Based on these results, it appears that curcumin, particularly nanocurcumin was effective against osmotic stress caused by hyperglycemia.

**Table 2:** The effect of curcumin and nanocurcumin on aldose reductase (AR) activity and sorbitol levels in rat lens.

**Groups**	**AR**	**Sorbitol**
Control	52.93 ± 5.14	0.318 ± 0.060 ^a^
Diabetic (D)	56.29 ± 3.79	2.671 ± 0.311^b^
D + Curcumin	53.46 ± 12.75	2.178 ± 0.162 ^c^
D + Nanocurcumin	50.65 ± 4.21	1.589 ± 0.141 ^d^
D + Blank particles	56.12 ± 14.77	2.442 ± 0.102 ^e^
	p=0.938	p=0.000

The data are mean ± SD (n=4). Different superscripts ^a, b, c, d, e^) indicate data are significantly different from other groups. AR activity is expressed as µ moles NADPH oxidized/h/100 mg protein and sorbitol levels as µ moles/g lens.

**Table 3:** The effect of curcumin and nanocurcumin on protein content of rat lens.

**Groups**	**Total protein (mg/g lens**)	**Soluble protein (mg/g lens)**	**Soluble protein (%**)
Control	498 ± 21.63^a^	338 ± 16.54^a^	67.87
Diabetic (D)	318 ± 18.36 ^b,c^	126 ± 23.39 ^b^	39.62
D + Curcumin	366 ± 10.12 ^b,c^	183 ± 17.58 ^b^	50.00
D + Nanocurcumin	377 ± 60.09 ^b^	227 ± 42.57 ^c^	60.00
D + Blank particles	313 ± 28.59 ^c^	136 ± 19.43^b^	43.45
	p=0.001	p=0.001	

The data are mean ± SD (n=6). Different superscripts ^a,b,c)^ indicate that data are significantly different from other groups.

### Protein glycation

**Figure 4:**
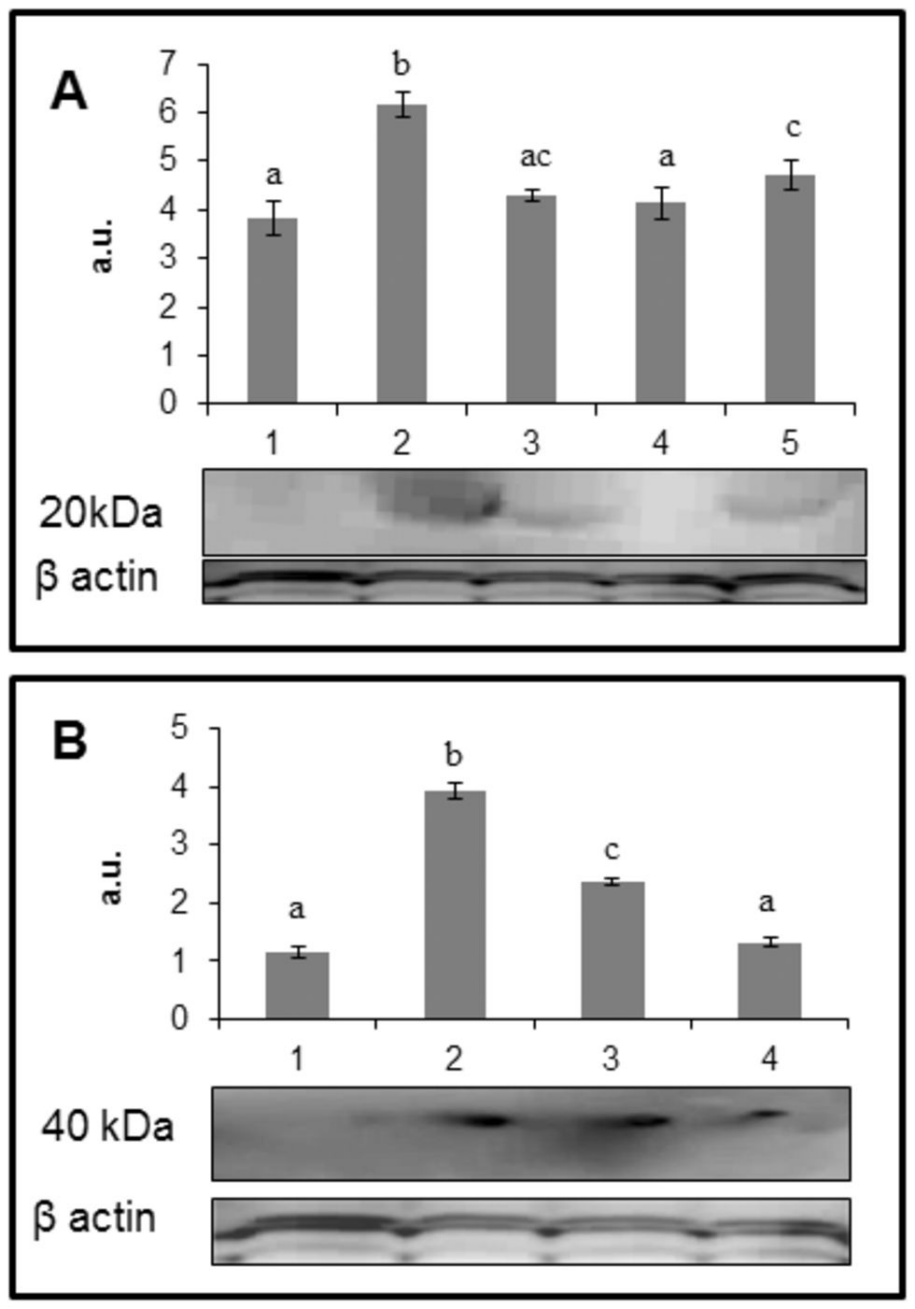
Immunodetection of advanced glycation endproducts, CML (Panel A) and AGE-BSA (Panel B), in the soluble protein fraction of rat lens. The data in bar diagram are mean ± SD (n=3) and the blot is a representative of three independent analyses. *Lane 1*: control; lane *2*: diabetic (D); lane *3*: D + curcumin; lane *4*: D + nanocurcumin and lane *5*: D + blank particles. Different superscripts indicate data are significantly different from other groups.

To understand whether curcumin feeding to diabetic rats could reduce protein glycation, we monitored a few prominent AGE in the lens by immunodetection using specific polyclonal antibodies. While immunoreactivity of glucose derived AGE, CML and AGE-BSA, in the soluble portion was undetectable in control rat lens, these two AGE detection was significant in untreated diabetic rat lens (Figure 4). Feeding of rats with curcumin and nanocurcumin significantly reduced the formation of AGE in soluble protein fraction and the effect with nanocurcumin was more prominent than curcumin (Figure 4). 

### Crystallin distribution, protein aggregation & insolubilization

Whatever the underlying mechanism, alterations in protein profile, and insolubilization of otherwise soluble protein, have been considered to be the ultimate changes that result in lens opacification. Therefore, we have analyzed the total, soluble and insoluble protein content in all the groups. There was a significant decrease in both total as well as soluble protein (p<0.001) in diabetic rats compared to control group (Table 3). Generally during progression and subsequent maturation of cataract, while there is a leakage of proteins from the lens due to osmotic stress in addition to degradation of lens proteins which is the main cause of decreased total protein content, there is an insolubilization of available soluble protein due to mechanisms mentioned above. Feeding the rats with curcumin improved the total and soluble protein levels (p<0.001), which is in agreement with the delay of onset of cataract in this group (Table 3). However, it should be noted that the improvement in percentage of soluble protein with nanocurcumin was remarkable compared to curcumin (Table 3).

**Figure 5:**
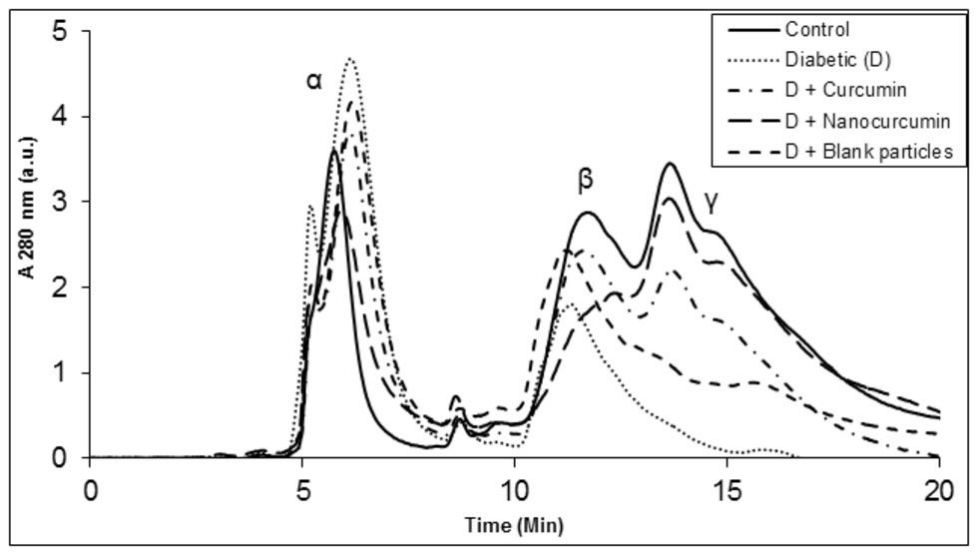
The effect of curcumin and nanocurcumin on crystallin distribution in the soluble protein fraction of rat lens. Soluble protein was loaded on TSK-G3000 SWXL gel filtration HPLC column and protein peaks were detected at 280 nm. Peaks representing α-, β- and γ- crystallins are indicated at their respective positions.

**Figure 6:**
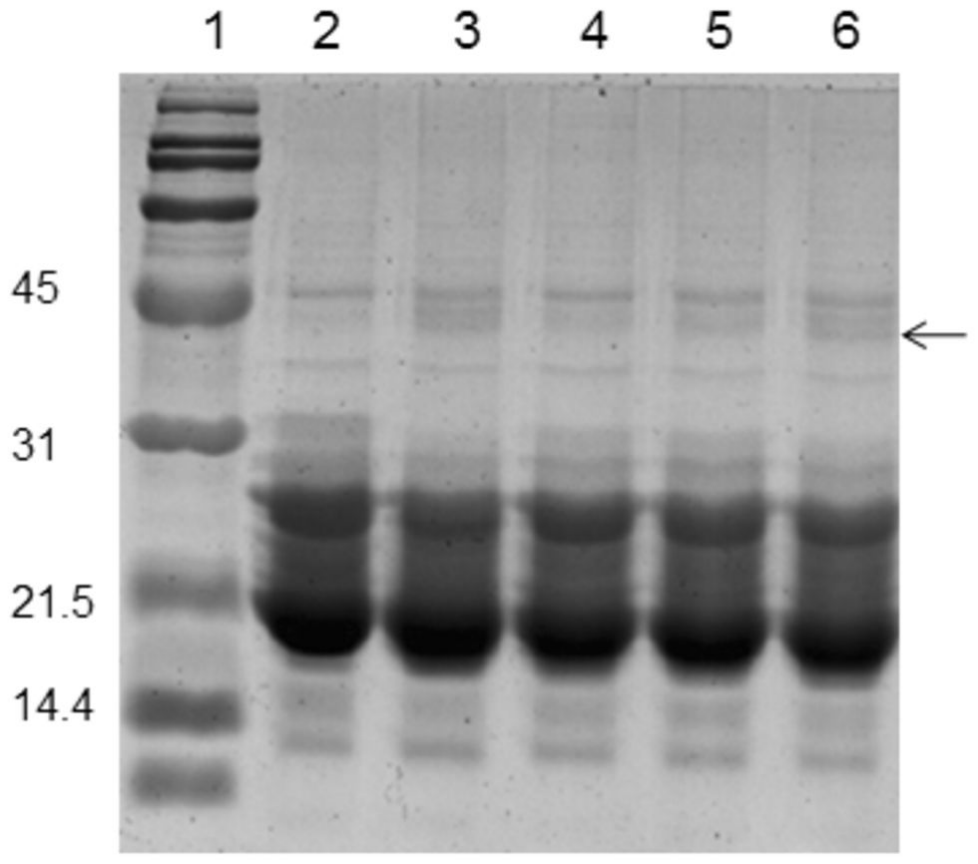
The effect of curcumin and nanocurcumin on protein crosslinking and the aggregation of the soluble fraction of the lens. Soluble protein was loaded onto a polyacrylamide gel. *Lane 1*: molecular mass markers; lane *2*: control group; lane *3*: diabetic group (D); lane *4*: D + curcumin group; lane *5*: D + nanocurcumin group and lane *6*: D + blank particles group. *Arrow*: cross-linked proteins.

To further investigate possible alterations in crystallin profile, the soluble proteins were analyzed by size exclusion chromatography. The distribution profile evidenced by HPLC showed a decrease in β- and α-crystallin abundance while there was an increase in α-crystallin peak in addition to appearance of a high molecular weight (HMW) aggregates peak in the void volume in diabetic untreated compared to control (Figure 5). The decrease in β- and γ-crystallins suggest protein modifications/degradation in diabetic cataract lens, which may be involved in the formation of HMW aggregates (including increase in α-crystallin peak) due to either cross-linking or aggregation or other modifications (Figure 5). The curcumin group showed improved crystallin distribution pattern and nano-curcumin showed crystallin profile comparable to that of control rats (Figures 5). Further, we have also monitored the cross-links of lens proteins and high molecular weight (HMW) aggregates by SDS-PAGE. The SDS-PAGE pattern of soluble protein showed an increased proportion of cross linked and aggregated proteins with the appearance of non-disulfide dimers of molecular mass 45 kDa in the diabetic rat lens compared to that of control and both the curcumin groups matched control group (Figure 6). 

## Discussion

In this study, a modified and highly reproducible method was employed to make curcumin encapsulated nanoparticles containing approximately 4.2 mg of curcumin per 50 mg polymer (PLGA). Our previous studies tested nanoparticles ability to improve peroral bioavailability of curcumin at least by 9 fold compared to that of plain curcumin [25]. Large amounts of curcumin encapsulated nanoparticles and curcumin free nanoparticles (~280 nm) were prepared and were freeze dried. The nanocurcumin was examined as a pharmacological intervention to retard the development and progression of cataract in an experimental model of diabetes with appropriate controls. Overall, there were significant beneficial effects of chronic nanocurcumin therapy on delaying the cataract in diabetic rats. The delay in progression of diabetic cataract by nanocurcumin was characterized by its ability to modulate biochemical mechanisms and pathways that are linked to the development of cataract such protein insolubilization, oxidative stress, protein glycation, crystallin distribution and polyol pathway. The beneficial effects of nanocurcumin on these biochemical pathways are distinctly superior compared to curcumin.

Nanocurcumin was effective than curcumin in delaying the diabetic cataracts in rats, although it could not prevent diabetic cataract completely at 2 mg/day dose. For example, cataract matured to stage 4 by 10 weeks in untreated diabetic rats while the cataract matured to 3.33 and 2.60 in curcumin and nanocurcumin treated diabetic rats respectively. The enhanced performance of nanocurcumin when compared to curcumin can be attributed to its improved oral bioavailability. However, it is very difficult to quantify using standard HPLC method that we employed for bioavailability ^25^ with such smaller doses (2 mg/ day) used in the present study. Therefore, efforts are underway to develop a sophisticated method such as LC-MS method to study the steady state plasma levels. Further, a dose response needs to be established in cataract and beyond.

Curcumin has been identified as the active principle of turmeric and has been shown to exhibit antioxidant, anti-inflammatory, antimicrobial, and anticarcinogenic activities [27,28].Curcumin was shown to induce apoptosis in human retinal endothelial cells and decrease VEGF release into media in vitro [29] and it also inhibits diabetes-induced elevation of serum VEGF levels in rat [30]. We have also demonstrated that supplementation of curcumin rescued photoreceptor degeneration in transgenic rats with P23H rhodopsin mutation [31].

Hence, earlier we have investigated the potential of curcumin to prevent the secondary complications of diabetes such as cataract and found that it delays diabetes-induced cataract in rats [12]. Nevertheless, curcumin at the dose (0.01%) equivalent or slightly higher than the amount of average daily intake (ADI) of turmeric could not prevent diabetic cataract completely. While increase in the dose (>0.01%) could not prevent diabetic cataract completely in STZ-diabetic rat model, the same dose in fact enhanced the cataract formation in galactose-rat model [32].

The major drawback of curcumin is poor physicochemical and biopharmaceutical properties leading to their low oral bioavailability from conventional dosage forms. To date, a number of studies have tried to elucidate the pharmacokinetics of curcumin, as it is poorly absorbed from the gastrointestinal tract after oral administration due to its low water solubility and low stability against gastrointestinal fluids and/or alkali/higher pH conditions. Further, rapid plasma clearance and conjugation of curcumin limit pharmacological exploitation of curcumin. Together, these characteristics lead to an unacceptably low oral bioavailability [17]. 

Therefore, numerous efforts have been undertaken to increase systemic bioavailability of curcumin which includes mainly complexing it with other substances. A study reported enhanced absorption and bioavailability of liposome-encapsulated curcumin by oral administration [33]. Further, this study significantly correlated plasma curcumin concentration with plasma antioxidant activities. Another study demonstrated that curcumin-loaded poly (lactide-co-glycolic acid) PLGA nanoparticles formulation results in enhanced cellular uptake, and increased bioactivity in vitro and superior bioavailability in vivo over curcumin [34]. Of the many approaches, nanoparticle-based delivery systems will probably be suitable for highly hydrophobic agents like curcumin circumventing the pitfalls of poor aqueous solubility, however further investigations are needed to understand the ability of nanoparticles in delivering very high dose compounds such as curcumin. Use of polymeric nanoparticles has been actively explored as oral delivery vehicles for pharmaceutically challenging compounds [23,24]. 

The oral dose (2 mg/day/rat) of curcumin and nanocurcumin used in this study corresponds to approximately 0.01% curcumin in the diet as used in our previous study [12]. The results with nanocurcumin in terms of cataract delay were encouraging and also indicate that one may use a higher dose of encapsulated nanocurcumin which might prevent or delay cataract to greater extent. In summary, the results of the present indicate that ingestion of nanocurcumin leads to improved oral bioavailability and thereby better efficacy compared to curcumin in delaying diabetic cataract in rats. Thus the present study paves way for further investigations of nanocurcumin as a potential pharmacological intervention for diabetic complications. 

## References

[1] (2011) Internal Diabetes Federation, Diabetes Atlas 5th edition.

[2] Michael J, Fowler MD (2008) Microvascular and Macrovascular Complications of Diabetes. Clin Diabetes 26: 77-82. doi:10.2337/diaclin.26.2.77.

[3] Congdon NG, [!(surname)!], Lietman T (2003) Important Causes of Visual Impairment in the World Today JAMA 290: 2057-2060. doi:10.1001/jama.290.15.2057. PubMed: 14559961. 14559961

[4] Brownlee M (2001) Biochemistry and molecular cell biology of diabetic complications. Nature 414: 813-820. doi:10.1038/414813a. PubMed: 11742414. 11742414

[5] Gabir MM, Hanson RL, Dabelea D, Imperatore G, Roumain J et al. (2000) Plasma glucose and prediction of microvascular disease and mortality: evaluation of 1997 American Diabetes Association and 1999 World Health Organization criteria for diagnosis of diabetes. Diabetes Care 23: 1113-1118. doi:10.2337/diacare.23.8.1113. PubMed: 10937507. 10937507

[6] Spector A (2000) Review: Oxidative stress and disease. J Ocul Pharmacol Ther 16: 193-201. doi:10.1089/jop.2000.16.193. PubMed: 10803430. 10803430

[7] Kyselova Z, Stefek M, Bauer V (2004) Pharmacological prevention of diabetic cataract. J Diabetes Complications 18: 129-140. doi:10.1016/S1056-8727(03)00009-6. PubMed: 15120709. 15120709

[8] Mares JA (2004) High-dose antioxidant supplementation and cataract risk. Nutr Rev 62: 28-32. doi:10.1301/nr.2004.jan.28-32. PubMed: 14995054. 14995054

[9] Taylor A, Jacques PF, Epstein EM (1995) Relations among aging, antioxidant status, and cataract. Am J Clin Nutr 62: 1439S-1447S. PubMed: 7495245. 7495245 10.1093/ajcn/62.6.1439S

[10] Kumar PA, Reddy PY, Srinivas PN, Reddy GB (2009) Delay of diabetic cataract in rats by the antiglycating potential of cumin through modulation of alpha-crystallin chaperone activity. J Nutr Biochem 20: 553-562. doi:10.1016/j.jnutbio.2008.05.015. PubMed: 18789666. 18789666

[11] Saraswat M, Suryanarayana P, Reddy PY, Patil MA, Balakrishna N et al. (2010) Antiglycating potential of Zingiber officinalis and delay of diabetic cataract in rats. Mol Vis 16: 1525-1537. PubMed: 20806076. 20806076 PMC2925903

[12] Suryanarayana P, Saraswat M, Mrudula T, Krishna TP, Krishnaswamy K et al. (2005) Curcumin and turmeric delay streptozotocin-induced diabetic cataract in rats. Invest Ophthalmol Vis Sci 46: 2092-2099. doi:10.1167/iovs.04-1304. PubMed: 15914628. 15914628

[13] Suryanarayana P, Saraswat M, Petrash JM, Reddy GB (2007) Emblica officinalis and its enriched tannoids delay streptozotocin-induced diabetic cataract in rats. Mol Vis 13: 1291-1297. PubMed: 17679931. 17679931

[14] Mrudula T, Suryanarayana P, Srinivas PN, Reddy GB (2007) Effect of curcumin on hyperglycemia-induced vascular endothelial growth factor expression in streptozotocin-induced diabetic rat retina. Biochem Biophys Res Commun 361: 528-532. doi:10.1016/j.bbrc.2007.07.059. PubMed: 17662242. 17662242

[15] Muthenna P, Suryanarayana P, Gunda SK, Petrash JM, Reddy GB (2009) Inhibition of aldose reductase by dietary antioxidant curcumin: mechanism of inhibition, specificity and significance. FEBS Lett 583: 3637-3642. doi:10.1016/j.febslet.2009.10.042. PubMed: 19850041. 19850041

[16] Krishnaswamy K, Raghuramulu N (1998) Bioactive phytochemicals with emphasis on dietary practices. Indian J Med Res 108: 167-181. PubMed: 9863273. 9863273

[17] Anand P, Kunnumakkara AB, Newman RA, Aggarwal BB (2007) Bioavailability of curcumin: problems and promises. Mol Pharm 4: 807-818. doi:10.1021/mp700113r. PubMed: 17999464. 17999464

[18] Sharma RA, Steward WP, Gescher AJ (2007) Pharmacokinetics and pharmacodynamics of curcumin. Adv Exp Med Biol 595: 453-470. doi:10.1007/978-0-387-46401-5_20. PubMed: 17569224. 17569224

[19] Pan MH, Huang TM, Lin JK (1999) Biotransformation of curcumin through reduction and glucuronidation in mice. Drug Metab Dispos 27: 486-494. PubMed: 10101144. 10101144

[20] Sharma RA, Euden SA, Platton SL, Cooke DN, Shafayat A et al. (2004) Phase I clinical trial of oral curcumin: biomarkers of systemic activity and compliance. Clin Cancer Res 10: 6847-6854. doi:10.1158/1078-0432.CCR-04-0744. PubMed: 15501961. 15501961

[21] Kumar Ravi (2012) Can efficient delivery systems leverage benefits of antioxidants leading to potential medicines? Drug Discov Today 17: 407-408. doi:10.1016/j.drudis.2012.01.024. PubMed: 22326671. 22326671

[22] Grama CN, Ankola DD, Ravi Kumar MNV (2011) Poly(lactide-co-glycolide) nanoparticles for peroral delivery of bioactives. Curr Opin_Colloid Interface Sci 16: 238-245. doi:10.1016/j.cocis.2010.11.005.

[23] Italia JL, Sharp A, Carter KC, Warn P, Kumar MN (2011) Peroral amphotericin B polymer nanoparticles lead to comparable or superior in vivo antifungal activity to that of intravenous Ambisome(R) or Fungizone. PLOS ONE 6: e25744. doi:10.1371/journal.pone.0025744. PubMed: 21998690. 21998690 PMC3188565

[24] Mittal G, Carswell H, Brett R, Currie S, Kumar MN (2011) Development and evaluation of polymer nanoparticles for oral delivery of estradiol to rat brain in a model of Alzheimer's pathology. J Control Release 150: 220-228. doi:10.1016/j.jconrel.2010.11.013. PubMed: 21111014. 21111014

[25] Shaikh J, Ankola DD, Beniwal V, Singh D, Kumar MN (2009) Nanoparticle encapsulation improves oral bioavailability of curcumin by at least 9-fold when compared to curcumin administered with piperine as absorption enhancer. Eur J Pharm Sci 37: 223-230. doi:10.1016/j.ejps.2009.02.019. PubMed: 19491009. 19491009

[26] Reddy PY, Giridharan NV, Reddy GB (2012) Activation of sorbitol pathway in metabolic syndrome and increased susceptibility to cataract in Wistar-Obese rats. Mol Vis 18: 495-503. PubMed: 22393276. 22393276 PMC3291519

[27] Aggarwal BB, Kumar A, Bharti AC (2003) Anticancer potential of curcumin: preclinical and clinical studies. Anticancer Res 23: 363-398. PubMed: 12680238. 12680238

[28] Anand P, Thomas SG, Kunnumakkara AB, Sundaram C, Harikumar KB et al. (2008) Biological activities of curcumin and its analogues (Congeners) made by man and Mother Nature. Biochem Pharmacol 76: 1590-1611. doi:10.1016/j.bcp.2008.08.008. PubMed: 18775680. 18775680

[29] Premanand C, Rema M, Sameer MZ, Sujatha M, Balasubramanyam M (2006) Effect of curcumin on proliferation of human retinal endothelial cells under in vitro conditions. Invest Ophthalmol Vis Sci 47: 2179-2184. doi:10.1167/iovs.05-0580. PubMed: 16639030. 16639030

[30] Kowluru RA, Kanwar M (2007) Effects of curcumin on retinal oxidative stress and inflammation in diabetes. Nutr Metab (Lond) 4: 8. doi:10.1186/1743-7075-4-8. PubMed: 17437639. 17437639 PMC1868028

[31] Vasireddy V, Chavali VR, Joseph VT, Kadam R, Lin JH et al. (2011) Rescue of photoreceptor degeneration by curcumin in transgenic rats with P23H rhodopsin mutation. PLOS ONE 6: e21193. doi:10.1371/journal.pone.0021193. PubMed: 21738619. 21738619 PMC3126808

[32] Suryanarayana P, Krishnaswamy K, Reddy GB (2003) Effect of curcumin on galactose-induced cataractogenesis in rats. Mol Vis 9: 223-230. PubMed: 12802258. 12802258

[33] Takahashi M, Uechi S, Takara K, Asikin Y, Wada K (2009) Evaluation of an oral carrier system in rats: bioavailability and antioxidant properties of liposome-encapsulated curcumin. J Agric Food Chem 57: 9141-9146. doi:10.1021/jf9013923. PubMed: 19757811. 19757811

[34] Anand P, Nair HB, Sung B, Kunnumakkara AB, Yadav VR et al. (2010) Design of curcumin-loaded PLGA nanoparticles formulation with enhanced cellular uptake, and increased bioactivity *in* *vitro* and superior bioavailability *in* *vivo* . Biochem Pharmacol 79: 330-338. doi:10.1016/j.bcp.2009.09.003. PubMed: 19735646. 19735646 PMC3181156

